# Prediction of appropriate ICD-therapy using infarct heterogeneity from CMR in patients with coronary artery disease

**DOI:** 10.1186/1532-429X-17-S1-P165

**Published:** 2015-02-03

**Authors:** Robert Jablonowski, Uzma Chaudhry, Henrik Engblom, Håkan Arheden, Einar Heiberg, Rasmus Borgquist, Marcus Carlsson

**Affiliations:** Cardiac MR group Lund, Dept of Clinical Physiology, Lund University, Lund, Sweden; Dept. of Biomedical Engineering, Faculty of Engineering, Lund University, Lund, Sweden; Dept. of Cardiology, Arrhytmia Clinic, Skane University Hospital, Lund University, Lund, Sweden

## Background

The heterogeneous peri-infarction zone surrounding the core infarct with cardiac magnetic resonance imaging (CMR) late gadolinium enhancement (LGE) has been linked to all-cause mortality in patients with coronary artery disease. Previously, the heterogeneity of fibrotic areas has been analyzed by threshold algorithms. We hypothesized that the heterogeneous peri-infarction zone is related to appropriate ICD-therapy in ischemic cardiomyopathy patients. Therefore, the purpose of this study was to investigate if 1) infarct heterogeneity can predict appropriate ICD-therapy and 2) evaluate which analysis method best depicts and quantifies the peri-infarction zone.

## Methods

**Figure 1:**
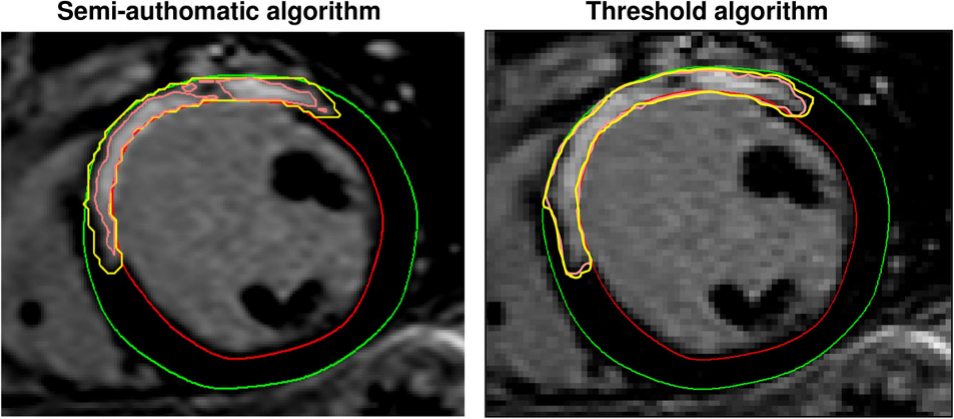
**Representative short axis LGE-CMR images from one patient evaluated for peri-infarction zone with the semi-automatic algorithm (left panel) and threshold algorithm (right panel).** The peri-infarction zone is defined as the area between the pink (infarct core) and yellow line. Red line=endocardium, green line=epicardium.

Ischemic cardiomyopathy patients with a primary prophylactic ICD who underwent CMR on a 1.5T scanner prior to ICD implantation were retrospectively included and divided into two groups (i) patients with appropriate ICD-therapy (anti-tachy pacing, shock or both) and (ii) patients with no ICD-therapy. A newly developed semi-automatic quantitative algorithm was used to evaluate the peri-infarction zone. This method was compared against a previously used threshold method with the total scar area defined as signal intensity (SI)>2SD from remote myocardium, infarct core as SI>3SD from remote and the peri-infarction zone defined as SI between 2 and 3SD from remote (Figure 1). Differences with a p<0.05 were considered statistically significant.

## Results

A total of 14 patients were included in the analysis, six patients with appropriate ICD-therapy (age 53±11years, 100% male, LV-EF 29±9%) and eight patients with no ICD-therapy (age 55±14years, 100% male, LV-EF 26±4%). The total scar burden was similar between both groups with and without ICD-therapy (49±13g vs 45±8g, p=0.1).

**Figure 2:**
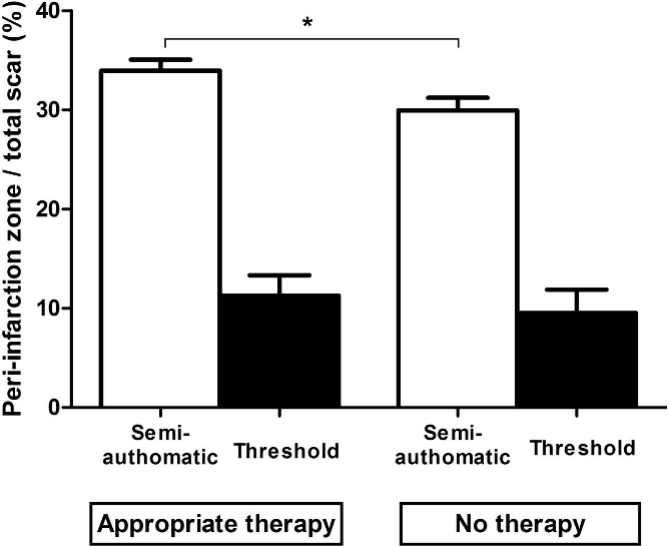
**Quantification of peri-infarction zone normalized to total scar in patients with and without appropriate ICD-therapy using a semi-automatic algorithm (white columns) and a threshold algorithm based on standard deviations (black columns).** The peri-infarction zone is significantly larger in patients with appropriate therapy compared to patients without therapy using the semi-automatic algorithm.

The mean peri-infarction zone normalized to the total scar using the semi-quantitative algorithm was larger in the group with appropriate therapy (34±1%) compared to the group with no ICD-therapy (30±1%, p=0.03), Figure 2. There was no difference between groups using the threshold algorithm for peri-infarction zone analysis (11±2% with appropriate ICD-therapy vs 10±2% with no therapy, p=0.4). There was a significant difference in peri-infarction zone normalized for total scar between the semi-automatic and threshold algorithm for patients with appropriate therapy (p=0.002) and no therapy (p=0.0003).

## Conclusions

The peri-infarction zone quantified on CMR using a semi-automatic algorithm was larger in patients with appropriate ICD-therapy compared to patients with no ICD-therapy. The use of a threshold algorithm did, however, not separate the groups. Accurate quantification and characterization of the peri-infarction zone could aid in the identification of patients with infarction and at risk of ventricular arrhythmias and help to improve patient selection for primary prevention with ICD-therapy.

## Funding

Lund University, Region of Scania.

